# Retraction of “Giant Thermal Switching via Phase Transition in MoTe_2_”

**DOI:** 10.1021/acs.jpclett.6c03015

**Published:** 2026-09-18

**Authors:** Zhuyao Chang, Nemo McIntosh, Zhao Liu, Riccardo Rurali

The authors retract this article (DOI: 10.1021/acs.jpclett.5c03836) due to concerns regarding the reliability of the presented data. Specifically, we have found errors in the calculation of the fourth-order IFCs for the 2*H* phase. We found out that 10–15 distorted supercells, out of the total 1024 for fourth-order IFCs of the 2*H* phase, converged correctly from the numerical point of view, but to a wrong minimum of the charge density. These few runs were sufficient to yield an artificially suppressed lattice thermal conductivity for the 2*H* phase, leading us to draw conclusions that we now know are not valid.

All of the results for the 1*T*′ phase are correct and could be reproduced. The results for the 2*H* phase using only second- and third-order IFCs are also correct and could be reproduced.

Based on these concerns, the overall findings of the work are unreliable, and the article is being retracted.

The original article was published on January 19, 2026, and retracted on September 18, 2026.

